# The Effects of Six Weeks Pulmonary Rehabilitation on Functional and Psychological Outcomes in Long-COVID Patients: Preliminary Results from Serbian Single Center Study

**DOI:** 10.3390/medicina60040671

**Published:** 2024-04-21

**Authors:** Natasa Mujovic, Dejan Nikolic, Filip Markovic, Mihailo Stjepanovic, Milica Zekovic, Hussain Saleh H. Ali, Dubravka Zivanovic, Milan Savic, Marija Laban

**Affiliations:** 1 Faculty of Medicine, University of Belgrade, 11000 Belgrade, Serbia; denikol27@gmail.com (D.N.); mihailostjepanovic@gmail.com (M.S.); dubravkazivanovic@yahoo.com (D.Z.); drmilansavic@gmail.com (M.S.); 2 Center for Physical Medicine and Rehabilitation, University Clinical Center of Serbia, 11000 Belgrade, Serbia; 3 Department of Physical Medicine and Rehabilitation, University Children’s Hospital, 11000 Belgrade, Serbia; 4 Clinic for Pulmonary Diseases, University Clinical Center of Serbia, 11000 Belgrade, Serbia; flp.mark@gmail.com (F.M.); marija.labanlazovic@gmail.com (M.L.); 5 Laboratory for Sports Medicine and Exercise Therapy, Institute of Medical Physiology “Rihard Burijan”, Faculty of Medicine, University of Belgrade, 11000 Belgrade, Serbia; milica.zekovic@med.bg.ac.rs; 6 King Faisal Medical City, Abha 62527, Saudi Arabia; xglxg@hotmail.com; 7 Clinic of Dermatology and Venerology, University Clinical Center of Serbia, 11000 Belgrade, Serbia; 8 Clinic for Lung Surgery, University Clinical Center of Serbia, 11000 Belgrade, Serbia

**Keywords:** long-COVID, physiology, psychology, rehabilitation

## Abstract

*Background and Objectives:* In this study, we aimed to evaluate the effects of six weeks of pulmonary rehabilitation on functional and psychological outcomes in long-COVID patients. *Material and Methods:* The prospective clinical study included 46 patients that were diagnosed with COVID-19. A respiratory rehabilitation program was implemented for six weeks. Further valuables were tested before the beginning of the rehabilitation program (admission) and six weeks after (discharge): SpO_2_, heart rate, respiratory rate, Visual Analogue Scale (VAS) score, Borg score, Sit-to-Stand (StS) test number of repetition, distance of 6-Minute Walking Test (6MWT), Patient Health Questionnaire (PHQ) 9 score and Generalized anxiety disorder (GAD) anxiety score. These parameters were tested before the rehabilitation program on admission and at discharge and after the rehabilitation program on admission and at discharge. The results were presented with standard descriptive and analytical methods. Differences between the continuous variables before and after physical rehabilitation intervention were tested using the Wilcoxon test. Graphical analysis is presented with a box plot. *Results:* On discharge, in comparison with admission, the values of SpO_2_ were significantly lower (*p* = 0.007) before the 6MWT, and VAS scores were significantly higher (*p* = 0.036), while after the 6MWT, VAS scores were significantly lower (*p* < 0.001) as were Borg scores (*p* = 0.016). On discharge, in comparison with admission, the respiratory rate was significantly higher (*p* = 0.005) before the StS test, and Borg scores were significantly lower (*p* = 0.001), while after the StS test, SpO_2_ levels were significantly higher (*p* = 0.036) and VAS scores were significantly lower (*p* < 0.001), as were Borg scores (*p* = 0.008). After discharge, the values of the StS test were significantly higher (*p* = 0.011), PHQ9 scores were significantly lower (*p* < 0.001) and GAD anxiety scores were significantly lower as well (*p* = 0.005), while the distances measured in meters on the 6MWT were significantly increased (*p* < 0.001). *Conclusions:* A structured rehabilitation program in our study was shown to have beneficial effects on physiological, psychological and functional improvements in patients with long-COVID, and therefore it is advisable for these patients.

## 1. Introduction

Long-COVID presents a condition that poses a significant burden to medicine and public health across the world [1]. Previously, long-COVID was described as present symptoms that last more than three months after the disease onset [1]. According to the World Health Organization (WHO), post-COVID was defined as a condition that occurs in individuals with a history of probable or confirmed SARS-CoV-2 infection, usually 3 months from the onset, and with symptoms that last for at least 2 months and cannot be explained by an alternative diagnosis [2]. In a recent study, it was pointed out that 31–69% of those who survived a COVID-19 infection will develop long-COVID symptoms [3]. The burden of long-COVID has been stressed previously, when it was noticed that, according to the WHO’s data, 17 million people in Europe are assumed to be living with long-COVID, while according to the Centers for Disease Control and Prevention (CDC), it is estimated that nearly every fifth American adult has long-COVID [4].

Long-COVID can be assumed as a multisystem disease [4]. Previously, it has been described that post-COVID syndrome involves the musculoskeletal, neurological, respiratory and digestive systems, including depression [5]. Endocrinal and reproductive systems’ involvement, as well as immune dysregulation, have also been noted in long-COVID [6].

In recent research, it has been pointed out that the female gender, increasing age and experiencing more than five symptoms in the acute stage of COVID-19 are associated with increased risk of long-COVID development [7]. Moreover, other risk factors were described as well such as smoking, comorbidities, socioeconomic deprivation and personal resilience level [8].

The rehabilitation of patients with long-COVID should include a multidisciplinary rehabilitation team [8]. According to the National Institute for Health and Care Excellence (NICE) guidelines, it is recommended to implement integrated multidisciplinary rehabilitation with the expertise of healthcare specialists in fatigue and respiratory symptom management, bearing in mind that additional expertise might be needed depending on symptoms and the age of the individual [9]. Moreover, it has been suggested that the rehabilitation of critical COVID-19 survivors with severe pulmonary or cardiac injury might not be suitable, and the proposal of certain exclusion criteria: resting heart rate > 100 beats/min., blood pressure < 90/60 mmHg or >140/90 mmHg, blood oxygen saturation below 95% or conditions where exercise is contraindicated [10]. However, in another study, it was stressed that for patients with long-COVID, particularly for those with cardiopulmonary pathology, significant rehabilitation might be needed for ability improvement to participate in the activities of daily living [11]. Furthermore, in the randomized controlled trial of Jimeno-Almazán et al., it was pointed out that for those with post-COVID long term disability, exercise could be beneficial due to improvements in cardiovascular and strength fitness, mood disorder symptomatology and quality of life [12].

In this study, we aimed to evaluate the effects of six weeks of pulmonary rehabilitation on functional and psychological outcomes in long-COVID patients.

## 2. Methodology

### 2.1. Study Group

The prospective clinical study included 46 patients that were diagnosed with COVID-19 and who were referred 6 months after COVID-19 resolution to the Clinic for Pulmonary Diseases at the University Clinical Center of Serbia for outpatient rehabilitation due to presence of extended symptoms of COVID-19 including dyspnea and fatigue. All patients were assessed by Board-certified Internal Medicine specialists and Board-certified Physical Medicine and Rehabilitation specialists. Further variables were analyzed: age, gender, body mass index, length of outpatient hospitalization, SpO_2_ was analyzed twice, first on initial hospitalization due to the COVID-19 infection and on admission to outpatient rehabilitation program, need for oxygen support, computerized tomography (CT) score, presence of respiratory insufficiency, malignancy, obesity, respiratory, cardiovascular, endocrinological and psychiatric comorbidities, diabetes mellitus and its complications. Complications that were evaluated included pulmonary thromboembolism, pneumomediastinum, pneumothorax and sepsis. Additionally, duration of applied physical therapy on initial hospitalization due to the COVID-19 infection was analyzed as well as mean for CURB-65.

Exclusion criteria for the study participants were presence of absolute (unstable angina and myocardial infarction during previous month) or relative (resting heart rate more than 120, and regarding blood pressure (BP) more than 180 mmHg for systolic and more than 100 mmHg for diastolic BP) contraindications for 6MWT [13,14], and presence of chronic obstructive pulmonary disease (COPD).

The eligible participants were asked at discharge from outpatient rehabilitation program further question: have you taken antidepressant or anxiolytic medications before admission and during outpatient rehabilitation program? None of the participants from study group had taken antidepressant or anxiolytic medications.

All participants were informed prior to inclusion in this study and consent was obtained. This study followed the principles of good clinical practice and Declaration of Helsinki. The Institutional Review Board approved the study (Date: 31 March 2022; Number 695/6).

### 2.2. Study Instruments

For the evaluation of functional therapeutic response, we used 6-Minute Walking Test (6MWT) that provides prognostic data in patients with pulmonary and cardiac diseases [15]. It is a self-paced test and measures the distance walked in 6 min in meters [13]. Preparation for the test was performed in accordance with the European Respiratory Society and American Thoracic Society [13,16]. If necessary, patients can stop and use oxygen in cases where needed [13]. Further symptoms were indications for stopping the 6MWT: presence of chest pain, intolerable dyspnea, leg cramps, staggering, diaphoresis as well as pale or ashen appearance [14].

Sit-to-Stand (StS) test is a functional performance measure where time taken to stand from seated position is measured as well as number of repetitions in certain time period [17]. This test measures lower limb muscle strength [18]. In this study, we performed one-minute StS test. We followed the standard for chair height of 46 cm and StS test was performed without arm rests [19].

Visual Analogue Scale (VAS) was used to assess fatigue. VAS uses 100 mm in length scale. As reported in previous studies, we used centimeters with a range from 0 to 10 [20]. It is a self-reported scale [21]. Patients were asked to point to the place on the scale that best describes the pain intensity.

Borg Scale was used to assess breathlessness during exercise. We used modified Borg scale as a 10-point scale [22].

A 9-item Patient Health Questionnaire (PHQ) was used to evaluate depression with a score range from 0 to 27. Every item from PHQ9 was scored from 0 (not at all) to 3 (nearly every day) [23].

The CURB-65 is a 6-point scale in which further parameters are measured: confusion, urea > 7 mm/L, respiratory rate ≥ 30/minute, low blood pressure (systolic < 90 mm Hg and diastolic ≤ 60 mm Hg) and age ≥ 65 years [24].

Generalized anxiety disorder (GAD) is a 7-item scale that was used to assess anxiety in study group of participants with a total score ranging from 0 to 21 [25].

After the 6MWT, patients filled in the psychological tests for about 45 min and then were instructed to perform StS.

### 2.3. Rehabilitation Protocol

Physical therapy and rehabilitation program was implemented twice in these patients. The first administration of physical therapy and rehabilitation was on initial hospitalization due to the COVID-19 infection, and duration, expressed in days, was taken from the medical history of the patients from their medical records.

The second physical therapy and rehabilitation treatment was implemented on first visit (admission) when patients were screened and included in the outpatient rehabilitation program. After the initial screening, patients were included into the respiratory rehabilitation program for six weeks (five days per week) that consisted of ipratropium bromide inhalation as introductory procedure, then breathing exercise and exercises for chest expansion followed by the 10 min stationary bicycle cycling and treadmill for a duration of 10 min with a speed of 4 km per hour. The entire program duration was 45 min per daily visit.

Breathing exercises included two components (inhalation and exhalation). Inhalation was done through the nose and exhalation was done through the mouth. Breathing exercises were repeated 10 times per daily visit.

Chest expansion exercises comprised active arm movements in shoulder region: abduction, adduction and extension followed by profound inhalation and exhalation, with 10 repetitions per daily visit.

Further valuables were analyzed on admission and at discharge from the outpatient rehabilitation program on two occasions for 6MWT and StS, before and after testing: SpO_2_, heart rate, respiratory rate, VAS score and Borg score. StS test number of repetition, distance of 6MWT, PHQ9 score and GAD anxiety score were tested on admission and at discharge from outpatient rehabilitation program.

### 2.4. Statistical Analysis

Statistical analysis was conducted using the IBM SPSS Statistics version 26.0 (IBM Corporation, Armonk, NY, USA) program. The results were presented as numbers (N) and percentages (%) for the categorical variables and mean values (MV) with a standard deviation (SD) or median (M) with interquartile range (IQR) for the continuous variables. The Shapiro–Wilk test was used to test the normality of data distribution. Differences between the continuous variables (SpO_2_, heart rate, respiratory rate, VAS and Borg score) before and after testing on two occasions separately, on admission and at discharge, from outpatient rehabilitation program for 6MWT and StS were tested by the Wilcoxon test. Differences between the continuous variables (SpO_2_, heart rate, respiratory rate, VAS and Borg score) between admission and discharge from outpatient rehabilitation program before 6MWT and StS, as well as after 6MWT and StS, were tested by the Wilcoxon test. Differences for StS test, distance of 6MWT, PHQ9 score and GAD anxiety score between admission and discharge from outpatient rehabilitation program were tested by the Wilcoxon test. The statistical significance was set at *p* < 0.05.

Missing data for any tested variable were less than 2%, so in case of missing data for a defined variable, these fields were left blank and not included in the analysis.

The power of this study was calculated with the help of G*Power software, version 3.1.9.4 (Kiel, Germany) and was based on standard statistical criteria, two-sided testing, *p* < 0.05 and effect size 0.987 for equal group sizes before and after the intervention. The t-test was obtained based on the literature data for the value of 6MWT before and after rehabilitation and a total of 36 patients were needed to obtain a statistically significant difference in the value of 6MWT before and after rehabilitation. In order to ensure the sample size, ten more patients were additionally included.

Graphical analysis was presented with a box plot. Box limits indicate the range of the central 50% of the data (the range between the 25th and 75th percentile), with a central line marking the median value. Lines extend from each box to capture the range of the remaining data (maximal–minimal values), with dots placed past the line edges to indicate outliers.

## 3. Results

In Table 1, the demographic and clinical characteristics of the patients are presented. The mean age of the participants was 54.87 ± 13.78 years, the median body mass index was 27.13 and 45.7% of the ones with respiratory insufficiency. The mean number of comorbidities was 2, and 14 (30.4%) of the evaluated patients had complications, where pulmonary embolism was noticed in 5 (10.9%) patients. Oxygen support was administered in 35 (76.1%) of the tested participants. The median value of SpO_2_ on admission to initial hospitalization for COVID-19 was 90.0%. The mean duration of physical therapy and rehabilitation during initial hospitalization due to COVID-19 infection was 35.5 days. The median value of CURB-65 was 1 (Table 1).

**Figure 1:**
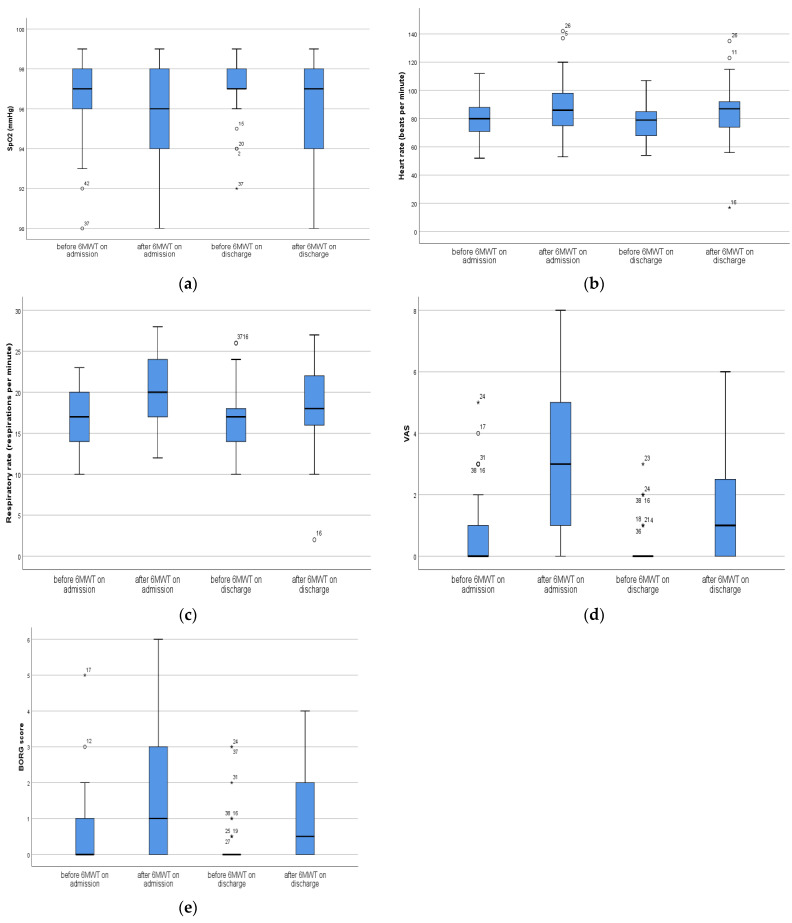
(**a**): Differences in SpO_2_ before and after 6MWT; (**b**): Differences in HR before and after 6MWT; (**c**): Differences in RR before and after 6MWT; (**d**): Differences in VAS before and after 6MWT; (**e**): Differences in Borg score before and after 6MWT. Box limits indicate the range of the central 50% of the data (the range between the 25th and 75th percentile), with a central line marking the median value. Lines extend from each box to capture the range of the remaining data (maximal-minimal values), with dots placed past the line edges to indicate outliers. Outliers, values they lie on either end of a data, are labeled with a * outside of the range of the whiskers in the box plot. Any data point further than within 1.5 times the interquartile range is considered an outlier. This study included 46 patients before and after intervention.

On admission, after the 6MWT heart rate (median value before was 80 and after 86), respiratory rate (median value before was 17 and after 20), the VAS score (median value before was 0 and after 3) and the Borg score (median value before was 0 and after 1) were significantly higher (*p* < 0.001) compared with before testing, while after discharge from the hospital after 6MWT further tested variables (heart rate (median value before was 79 and after 87), RR (median value before was 17 and after 18), the VAS score (median value before was 0 and after 1) and the Borg score (median value before was 0 and after 0.5)) were significantly higher (*p* < 0.001) compared with before testing, while for SpO_2_ (median value before was 97 and after 97) there was significant difference (*p* < 0.001) (Table 2, Figure 1a–e).

**Figure 2:**
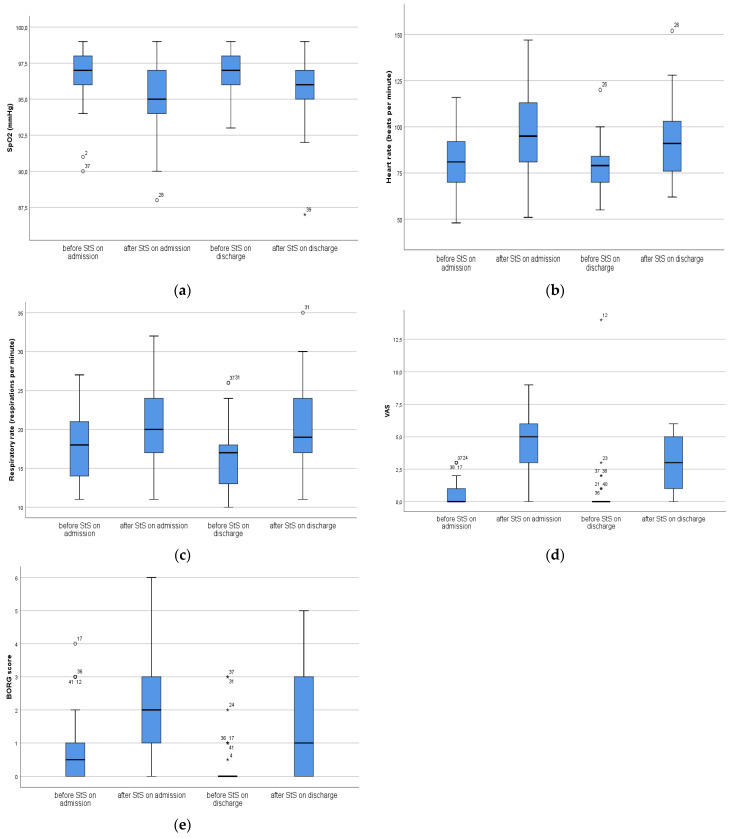
(**a**): Differences in SpO_2_ before and after StS; (**b**): Differences in HR before and after StS; (**c**): Differences in RR before and after StS; (**d**): Differences in VAS before and after StS; (**e**): Differences in Borg score before and after StS. Box limits indicate the range of the central 50% of the data (the range between the 25th and 75th percentile), with a central line marking the median value. Lines extend from each box to capture the range of the remaining data (maximal–minimal values), with dots placed past the line edges to indicate outliers. Outliers, values they lie on either end of a data, are labeled with a * outside of the range of the whiskers in the box plot. Any data point further than within 1.5 times the interquartile range is considered an outlier. This study included 46 patients before and after intervention.

Regarding StS test on admission from the hospital, heart rate (median value before was 81 and after 95), respiratory rate (median value before was 18 and after 20), the VAS score (median value before was 0 and after 5) and the Borg score (median value before was 0.5 and after 2) were significantly higher after the testing versus before the test (*p* < 0.001), while on SpO_2_ (median value before was 97 and after 95) was significantly lower after StS test compared with before the test (*p* < 0.001). At discharge from the hospital, heart rate (median value before was 79 and after 91), respiratory rate (median value before was 17 and after 19), the VAS score (median value before was 0 and after 3) and the Borg score (median value before was 0 and after 1) were significantly higher after the testing versus before the test (*p* < 0.001), while SpO_2_ (median value before was 97 and after 96) was significantly lower after StS test compared with before the test (*p* < 0.001) (Table 2, Figure 2a–e).

On discharge, compared with admission, the values of SpO_2_ were significantly lower (*p* = 0.007) before the 6MWT, and VAS scores were significantly higher (*p* = 0.036), while after the 6MWT, VAS scores were significantly lower (*p* < 0.001) as were Borg scores (*p* = 0.016) (Table 3).

On discharge, compared with admission, the respiratory rates were significantly higher (*p* = 0.005) before the StS test, and Borg scores were significantly lower (*p* = 0.001), while after the StS test, SpO_2_ levels were significantly higher (*p* = 0.036) and VAS scores were significantly lower (*p* < 0.001) and Borg scores as well (*p* = 0.008) (Table 3).

After discharge, the values of the StS test (median value on admission—24 and at discharge—25) were significantly higher (*p* = 0.011), PHQ9 scores (median value on admission—7 and at discharge—3) were significantly lower (*p* < 0.001) and GAD anxiety scores (median value on admission—2.5 and at discharge—2) were significantly lower as well (*p* = 0.005), while the distances measured in meters of the 6MWT (median value on admission—506 and at discharge—588) significantly increased (*p* < 0.001) (Table 4).

## 4. Discussion

In our study, the mean value of SpO_2_ for study patients on admission at initial hospitalization due to COVID-19 and prior inclusion into the rehabilitation program was 90%, and these were considered hypoxic [26]. Moreover, three out of four patients needed oxygen support, while somewhat less than half of the enrolled participants presented on admission with respiratory insufficiency. The mean value of the CURB-65 score of 1 in our study population indicated that these patients were at low risk of mortality [24] and thus could be included for referral for an outpatient rehabilitation program.

Our findings demonstrated that on admission to the rehabilitation program after the completion of 6MWT on first visit, there were significant increases in heart rates and respiratory rates as well as significant increases in the values of the VAS scores and Borg scores versus values just before starting the test. The same trend remained following discharge from the rehabilitation program, with the presence of significant changes in oxygen saturation as well. However, positive effects of 6MWT were noticed over the period of six weeks of the rehabilitation program in the studied group, where VAS scores were significantly lower and Borg scores were significantly lower as well after the 6MWT between admission and discharge. It should be noticed that over the six-week period of the rehabilitation program, the VAS score values before 6MWT differed significantly. Moreover, there were significant increases in the distances of 6MWT between admission and discharge from the pulmonary rehabilitation program. The positive effects of respiratory rehabilitation in patients with COPD were described in the study of Gosselink et al. [27] where the authors stated that there were increases in 6MWT distance and reductions in Borg scores. Furthermore, in the study of Dierckx et al. [28], it was noticed that three months of respiratory rehabilitation of long-COVID patients had positive effects in improving the distances of 6MWT and reductions in Borg scores. In a systematic review and meta-analysis of Ahmed et al. [29], the authors pointed out that pulmonary rehabilitation improves dyspnea and exercise capacity in patients with mild to severe symptoms in acute and chronic COVID-19. Our findings are in line with previous reports regarding the beneficials effects of pulmonary rehabilitation in post-COVID patients in terms of functional capacity improvement and the reduction of dyspnea.

Regarding fatigue, Kunoor et al. [30] demonstrated the beneficial effects of 4 weeks of pulmonary rehabilitation in patients with early postacute COVID disease. Moreover, Nopp et al. [31] stressed that a 6-week personalized interdisciplinary pulmonary rehabilitation program in patients with long-COVID had positive effects on fatigue. Finally, in the systematic review and meta-analysis of Oliveira et al. [32], it was noticed that pulmonary rehabilitation was associated with fatigue reduction in subjects with post-COVID-19 syndrome. However, it should be pointed out that in this study the fatigue assessment was conducted using the Fatigue Severity Scale [32], while in our study, we performed assessment via VAS. Our results corresponded with previous reports stressing the positive effects of pulmonary rehabilitation on fatigue reduction in patients with long-COVID.

In this study, we have demonstrated that on admission to the rehabilitation program, after the completion of an StS test on first visit, there were significant increases in heart rates and respiratory rates, significant decreases in oxygen saturation values and significant increases in the values of VAS and Borg scores compared with values just before starting the StS test. The same trend remained apparent on discharge from the rehabilitation program. It should be noticed that the positive effects of the StS test were noticed over the period of the six weeks of the rehabilitation program in the studied group, where the VAS and Borg scores were significantly lower after the StS test, while the oxygen saturation values were significantly higher between admission and discharge. Additionally, there were significant increases in the repetitions of the StS test between admission and discharge from the pulmonary rehabilitation program. The positive effects of the pulmonary rehabilitation program were noted in a study of Nair et al. [33], where the authors evaluated the effects of inpatient pulmonary rehabilitation on improvements to functional outcomes in patients with post-COVID-19 fibrosis. They pointed out that there were significant increases in the repetitions of StS tests in these patients [33].

Considering the effects of the six-week rehabilitation program from our study, we have shown that there were significant reductions in depression as well as anxiety in the evaluated patients. In a systematic review and meta-analysis of Martinez-Pozas et al. [34], it was demonstrated that pulmonary rehabilitation had significant positive effects on depression and anxiety in patients with long-COVID. Additional studies of Moine et al. [35] stressed that a four-week inpatient pulmonary rehabilitation of individuals with long-COVID symptoms had positive effects on reductions in anxiety and depression. The results of our study regarding depression and anxiety are consistent with previous findings, suggesting the importance of pulmonary rehabilitation program implementation in patients with long-COVID.

This study has several limitations. First, the sample size in this study is small and thus further investigations are needed on larger samples of participants. Another limitation is the single-center study. Study participants included members of the Serbian population; therefore, it is advisable to conduct additional studies on other populations, since possible intrinsic factors might, to the certain degree, influence the rehabilitation treatment outcomes for the tested variables. Also, the absence of a control group could be a limitation factor in this study.

## 5. Conclusions

An outpatient rehabilitation program in our study was shown to have beneficial effects on physiological, psychological and functional improvements in patients with long-COVID.

Outpatient pulmonary rehabilitation for post-COVID patients is advisable, beneficial and effective in the improvement of physical and functional outcomes. It has positive effects on patients’ psychological aspects such as a reduction in depression and anxiety. Therefore, the timely and adequate inclusion of these individuals in pulmonary rehabilitation programs will have positive impacts on personal well-being and improvements in overall quality of life as well as optimal social integration into society.

## Figures and Tables

**Table 1:** Demographic and clinical characteristics of patients.

Study Parameters	Values
Age (years), (MV ± SD)	54.87 ± 13.78
Gender: males/females, N(%)	26 (56.5%)/20 (43.5%)
Body mass index, M (25–75% IQR)	27.38 (25.67–32.75)
Outpatient hospitalization length (days), M (25–75% IQR)	14 (7–22)
SpO_2_ on admission on initial hospitalization due to COVID-19, M (25–75% IQR)	90.0 (83.5–96.0)
Oxygen support, N (%)	35 (76.1%)
CT score, M (25–75% IQR)	14 (11–18)
Respiratory insufficiency, N (%)	21 (45.7%)
Malignancy, N (%)	5 (10.9%)
Obesity, N (%)	8 (17.4%)
Diabetes mellitus, N (%)	9 (19.6%)
Diabetes mellitus complications, N (%)	4 (8.7%)
Number of comorbidities, M (25–75% IQR)	2 (1–2)
Pulmonary comorbidity, N (%)	12 (26.1%)
Cardiovascular comorbidity, N (%)	28 (60.8%)
Endocrinological comorbidities, N (%)	13 (28.3%)
Psychiatric comorbidities, N (%)	2 (4.3%)
Complications, N (%)	14 (30.4%)
Pulmonary thromboembolism, N (%)	5 (10.9%)
Pneumomediastinum, N (%)	4 (8.7%)
Pneumothorax, N (%)	2 (4.3%)
Sepsis, N (%)	3 (6.5%)
Physical therapy duration on initial hospitalization due to COVID-19 (days), M (25–75% IQR)	35.5 (25.5–47.75)
CURB-65, M (25–75% IQR)	1 (1–2)

MV—mean value; SD—standard deviation; M—median; N—number; IQR—interquartile range.

**Table 2:** Patients characteristics before testing and after testing for 6MWT and StS test on admission and at discharge from outpatient rehabilitation program.

	Outpatient Rehabilitation Program					
Tested Variables M (25–75% IQR)	Admission			Discharge		
	6 MWT					
	Before Testing	After Testing	*p* * Value	Before Testing	After Testing	*p* * Value
SpO_2_	97 (96–98)	96 (94–98)	0.083	97 (97–98)	97 (94–98)	<0.001
Heart rate	80 (71–88)	86 (74.5–99.5)	<0.001	79 (67.5–85.5)	87 (74–92)	<0.001
RR	17 (14–20)	20 (17–24)	<0.001	17 (13.5–18.5)	18 (16–22)	<0.001
VAS	0 (0–1)	3 (1–5)	<0.001	0 (0–0)	1 (0–2.5)	<0.001
Borg score	0 (0–1)	1 (0–3)	<0.001	0 (0–0.25)	0.5 (0–2)	<0.001
	**StS test**					
	**Before Testing**	**After Testing**	*p* * Value	**Before Testing**	**After Testing**	*p* * Value
SpO_2_	97 (96–98)	95 (94–97.5)	<0.001	97 (96–98)	96 (95–97)	<0.001
Heart rate	81 (68.5–92)	95 (81–113.5)	<0.001	79 (68.5–84.5)	91 (76–104)	<0.001
RR	18 (14–21.5)	20 (17–24)	<0.001	17 (13–18)	19 (17–24)	<0.001
VAS	0 (0–1)	5 (3–6)	<0.001	0 (0–0)	3 (1–5)	<0.001
Borg score	0.5 (0–1)	2 (1–3)	<0.001	0 (0–0)	1 (0–3)	<0.001

6MWT—6 minute walking test; StS test—Sit-to-Stand; RR—respiratory rate; VAS—visual analog scale; M—median; IQR—interquartile range; *—Wilcoxon test.

**Table 3:** Differences in tested variables between admission and discharge from outpatient rehabilitation program before and after testing.

	Outpatient Rehabilitation Program			
Tested Variables	Admission/Discharge		Admission/Discharge	
	6MWT		StS Test	
	*p* * Value before Testing	*p* * Value after Testing	*p* * Value before Testing	*p* * Value after Testing
SpO_2_	0.007	0.914	0.143	0.036
Heart rate	0.231	0.252	0.371	0.227
RR	0.103	0.097	0.005	0.293
VAS	0.036	<0.001	0.197	<0.001
Borg score	0.078	0.016	0.001	0.008

6MWT—6 min walking test; StS test—Sit-to-Stand; RR—respiratory rate; VAS—visual analog scale; *—Wilcoxon test.

**Table 4:** Changes in tested variables between admission and discharge.

Tested Variables M (25–75% IQR)	Outpatient Rehabilitation Program		
	Admission	Discharge	*p* * Value
StS test (repetitions)	24 (18–27.5)	25 (22–31.5)	0.011
Distance 6MWT (m)	506 (462–581)	588 (504–677)	<0.001
PHQ9 score	7 (4–10.25)	3 (1–5)	<0.001
GAD anxiety score	2.5 (1–5.5)	2 (0–3)	0.005

6MWT—6 min walking test; StS test—stand up and sit test; PHQ9—Patient Health Questionnaire 9; GAD—Generalized anxiety disorder; IQR—interquartile range; *—Wilcoxon test.

## Data Availability

The data presented in this study are available on reasonable request from the corresponding author.
